# Social Media and Health: Emerging Trends and Future Directions for Research on Young Adults

**DOI:** 10.3390/ijerph18158141

**Published:** 2021-07-31

**Authors:** Peng Wu, Ran Feng

**Affiliations:** Institute of Management Science and Engineering, Henan University, Kaifeng 475004, China; hfpjl789@163.com

**Keywords:** health-related social media, emotional support, information support, service quality

## Abstract

The ubiquity and affordances of social media have allowed young people to become both active posters and passive recipients of communication related to health. For instance, people may post exercise goals and behaviors on social media, while at the same time, they may be exposed to friends drinking alcohol and/or indulging in unhealthy snacking. This intersection of sociotechnical systems (i.e., social media), and health and wellbeing, has garnered increasing scholarly attention. How to understand and manage the continuous use intention of health-related social media, and then provide a better service platform and create a good service model for the needs of young adults has become an important topic in the research of social media and health-related fields. Based on the SOR theory, this paper constructs a theoretical model of factors affecting the continuous use intention of health-related social media. This paper uses questionnaires and structural equation empirical research methods, relevant software to process and analyze the data, and tests the applicability of the model. The results reveal that emotional support, information support, and service quality can significantly affect pan-family consciousness, pan-family consciousness can significantly affect the continuous use intention of health-related social media. In addition, our results also show that pan-family consciousness plays a mediating role between information support and the continuous use intention of health-related social media, pan-family consciousness plays a mediating role between the service quality and the continuous use intention of health-related social media, and self-efficacy plays a mediating role between pan-family consciousness and the continuous use intention of health-related social media. These findings have important implications for research and practice in the fields of the continuous use intention of health-related social media. We hope to help with the emerging trends and future directions for research on social media and health.

## 1. Introduction

The ubiquity and affordances of social media have allowed young people to become both active posters and passive recipients of communication related to health. For instance, people may post exercise goals and behaviors on social media, while, at the same time, they may be exposed to friends drinking alcohol and/or indulging in unhealthy snacking. This intersection of sociotechnical systems (i.e., social media), and health and wellbeing, has garnered increasing scholarly attention. How to understand and manage the continuous use intention of health-related social media, and then provide a better service platform and create a good service model for the needs of young adults, has become an important topic in the research of social media and health-related fields. With the rapid development of the internet, health-related social media that is characterized by sustainability, networking, and interactive participation, have flourished. Social media users can obtain health information and share personal health-related experiences through WeChat, QQ, microblog, question and answer platforms, online forums, and other social media. This health-related social media has become an important way for young people to pay attention to their health. The survey shows that 80% of young people are willing to share and communicate through health-related social media [1]. The World Health Organization defines the group under 44 as the young adult’s group. United Nations Educational, Scientific and Cultural Organization defines young adults as 14–34 years old [2,3]. The important difference between youth and other social groups is that youth has particularity and independence. With the rapid development of the social environment and network technology, health-related social media has become a new way of grouping and social organization [4]. How to understand and manage the continuous use intention of health-related social media, and then provide a better service platform and create a good service model for the needs of the young adults group, has become an important topic in the current research of the related field [5].

After combing the current related literature [6,7,8,9,10,11,12,13,14,15,16,17,18,19,20,21,22,23,24,25,26,27,28,29,30,31,32], we found that there is a lack of research on the continuous use intention of health-related social media. In particular, there is little literature on how the emotional experience of young adults in health-related social media affects the continuous use intention of health-related social media. Based on this, and according to the SOR (stimulus–organization–response) model, this paper takes social support and service quality as stimulus factors, and pan-family consciousness [33,34] as young adult’s emotional experiences, to study the mechanism of continuous use intention of health-related social media.

The contribution of this paper lies in the following: firstly, based on the SOR theory, this paper comprehensively considers the mechanism of social support and service quality on health-related social media continuance use intention, and constructs a theoretical model of the influencing factors of health-related social media continuance use intention. Second, although the study on the intention of the sustainable use of social media has attracted scholars’ attention, there are few studies on the intention of the sustainable use of health-related social media. This paper discusses how to promote the sustainable use intention of social media related to youth health, and provides new ideas for the research of social media and health. Third, although some scholars have discussed the mediating role of pan-family consciousness, there is a lack of research on the mediating role of pan-family consciousness on social support and service quality on health-related social media persistent use intention. This study proposes and tests the mediating effect of pan-family consciousness, and specifically reveals the important role of pan-family consciousness in promoting sustainable use intention.

## 2. Literature Review

### 2.1. SOR Model

The concept of the SOR model is derived from psychology, and is mainly used to explain the influence of the environment on human mental activities and behavior. The early psychologist Skinner [35] proposed the S–R model, based on the study of the relationship between the stimulus and the response, pointing out the relationship between the environment and behavior, but he regarded the model as a “black box” and ignored the individual—the true inner activity. Belk [36] also optimized the S–R model, adding an “O” variable, and proposed the SOR model, which is a stimulus–organism–response model, used mainly to study the impact of environmental variables on young adult behavior. Among the variables, S stands for stimulus, which can be the young adults’ factors, such as hobbies, family background, or motivation, and it can be an external environmental stimulus, such as political, economic, cultural, or another uncontrollable factor; it could also be an interpersonal factor, such as friend recommendations. External stimuli will have a certain impact on the subject, and will awaken the subject’s subconscious desire for a certain product or service. O stands for a cognitive organism, which refers to the inner activity of the subject after exposure to external stimuli, which is between the stimulus factors and the response behavior. R means that after the subject is stimulated, some inner activities result in a series of responses, such as adopting or avoiding, and accepting or rejecting. Mehrabin and Russell [37] proposed an environmental stimulus–organism–response model from the perspective of input–output, that is, the SOR model. It believes that humans are different from machines in the process of forming an organism. Later, Bitner [38] used the service environment as a background to study the impact of the physical environment on the young adult behavior of customers and employees, and specifically explained the psychological activities that are inherent in young adult shopping behavior; that is, external stimuli will cause young adults to produce cognitive, emotional, and even physical reactions, and these reactions will have an impact on the actual behavior of the young adults. Kotler [39] proposed a young adult behavior model based on the SOR model; the research explains the dynamic process of young adults from receiving external stimulus information to the external stimulus affecting their inner psychological activities, and then affecting their behavioral responses. It is believed that external environmental stimuli, such as marketing strategies, will first affect young adults’ awareness and cognition, and then young adults will make specific purchase decisions under the influence.

On the one hand, the SOR model is mainly applied to the field of consumer behavior. Howard and Sheth [40] proposed a young adult stimulus–response model, which believes that external factors have a certain impact on young adult behavior. In addition to controllable factors, such as product categories, product services, product prices, and service quality, there are also uncontrollable factors, such as the economy, politics, and culture. Other, more complex and diverse, factors can also be explained by the young adult stimulus–response model. Based on the environmental stimulus–organism–response model proposed by Mehrabian and Russell, Donovan and Rossiter [41] applied the SOR model to the marketing field, taking pleasure, control, and motivation as intermediate variables to study the influence of the store atmosphere on young adults’ shopping behavior. It is believed that if young adults have a more pleasant experience in the store, then they will spend longer in said store. Namkung and Jang [42] studied the impact of service fairness on young adults’ emotions and usage behavior under the SOR model. Lai [43] adopted the questionnaire survey method and structural equation modeling, using word-of-mouth communication as an external stimulus. From the micro- and macro-levels of the word-of-mouth effect, combined with young adults’ emotional perception and trust of word-of-mouth information, the influence of word-of-mouth on young adult buying behavior was investigated [44]. On the other hand, it is also applied to the study of the user behavior of information systems, such as online shopping. It has been found that the factors that stimulate young adults have changed greatly over time, and the factors that stimulate young adults have become numerous and complex. Eroglu [45] applied the SOR model to online shopping for the first time, using the shopping environment as a stimulus variable, young adult personality characteristics and emotional states as intermediary variables, and young adult behavior as a response variable, to build a model; the effect of environmental factors in online shops, on customer emotion and cognition, was then studied. It was found that if young adults have clear goals and rely on real product information to make purchases, then they are committed young adults. If young adults pay more attention to the shopping atmosphere and environment, then they environmentally impact young adults, and when the online shopping environment is too serious and boring, they will react accordingly—that is, they will abandon the purchase. The procedure of young adult’s information processing is the process of their inner psychological state. In this flow path, emotional factors play a very important role. Chang and Hsu [46] found that the information quality, system quality, and service quality of a website have a significant impact on young adults. The higher the information quality, system quality, and service quality of the website, the higher the young adults’ behavior and trust in said website. Jeong et al. [47], to verify whether Pine and Gilmore’s 4ES theory applies to online shopping websites, regarding product features as external stimulus factors, young adults’ sense of escapism and pleasure was regarded as an organism, and young adults’ browsing of websites was regarded as a reaction factor. It was found that, similarly to traditional young adult shopping behavior, features such as product display in online stores affect young adults’ pleasure and purchase intention. The more the product displays conform to the young adult’s psychology, the more enhanced the young adult’s pleasure and willingness to shop will be. Wang and Chang [48] used to search information as an external stimulus, and through an empirical investigation on the social platform Facebook, the influence of factors such as product risk perception and network social relationship strength on young adults’ purchase intention was discussed. The study found that for products with high-risk perceptions, the strength of the relationship with society can effectively affect young adults’ willingness to buy. If a product is recommended by people who are closer to the young adult’s social relationship, their acceptance and recognition will be higher. AI [49], based on the framework of the SOR model, studied the impact of promotions and recommendations on young adults’ purchase intentions in an online shopping environment, concerning the two aspects of cognition and emotion. It was found that there are differences in the influence of recommendations and promotions on young adults’ purchase intentions. In a recommendation context, young adults are relatively more rational and pay more attention to the experience during the shopping process; meanwhile, in the promotion context, the arousal factors in young adult emotions affect the willingness to purchase. The stronger the arousal factors, the stronger the impact on shopping behavior [50]. The SOR model has been widely used in consumer behavior and information system usage behavior. Therefore, this paper draws on the above-mentioned existing research and uses it as the theoretical basis for studying the continuous use intention of health-related social media.

### 2.2. Research on the Continuous Use Intention of Health-Related Social Media

Continuous use intention refers to the user’s willingness to continue to use a system in the future. Bhattacherjee [6] took the emerging B2C e-commerce website as the research object, and proposed a sustainable use model of information systems. This view of Bhattacherjee’s has aroused the attention and research of many scholars. According to their research objects, domestic and foreign scholars, on the basis of Bhattacherjee, have defined and optimized this model. Zhou [7] perfected it as the long-term sustainable use of an information system by young adults, who continued to use the information system for some time. The continuous use intention of social media may be due to a subjective choice, to a habitual difficulty to change, or to other reasons, which should be covered in the scope of the continuous use intention of social media. Yin et al. [8], in light of their continuous research on the use of social software, believe that the continuous use intention of social media refers to the continuous use of a certain software by young adults, rather than giving up use, or willingness to use other related alternatives [9]. Currently, in the field of continuous use intention of social media, Ku et al. [10] found, through research, that on social networking sites, subjective norms, privacy issues, satisfaction, and perceived critical quality all have an impact on the continuous use intention to use said sites. Benyoucef [11] used the theory of use and satisfaction to study the factors affecting the continuous use intention to use this site, and, through verification, found that young adults’ attitudes towards, and satisfaction with, social networking sites have a positive and significant impact on the continuous use intention to use them, while the perceived entertainment has an indirect effect. Lankton et al. [12] combined the trust theory and rational behavior theory to construct a theoretical model about the sustainable influence of attitudes, trust, and subjective norms on social networking site young adults’ willingness to use certain sites, also explaining how habit and trust explain and predict the continuous use intention to use in the context of new social networking sites. Shen [13] found, in an empirical study on the young adults of virtual websites, that interpersonal relationships are an important factor that determines the continuous use intention of social media, thus confirming the expressive effect of a social–emotional connection on online young adult psychology and young adult behavior. Hong et al. [14] studied the important factors that affect young adults’ behavior in enjoying digital reading services in a mobile context, and found that, relative to young adults’ cost considerations, the perceived usefulness of content is a key factor affecting the continuous use intention. Chen et al. [15] expanded and optimized the model from the aspects of microblog system quality, its characteristics, and subjective norms, based on the expectation confirmation theoretical model; in this way, the factors affecting the continuous use intentions were studied. Hrastinski [16] combined the expectation confirmation theory with the characteristics of social networks, and analyzed the factors affecting the continuous use intention to use online website content; it was found that content-driven perceived interaction, perceived usefulness, and perceived interest have significant positive effects on the continuous use intention to use online websites. Skadberg et al. [17], with the help of the expectation confirmation theory, found that the introduction of social presence and flow experience verifies that perceived usefulness and satisfaction have a significant impact on the continuous use intention to use WeChat, while immersive experience and social presence have an indirect impact on the continuous use intention to use WeChat. In addition, Jansson et al. [18], based on the innovation diffusion theory, young adult experience, and technology acceptance model, introduced several factors, such as perceived entertainment, young adult innovation, and peer influence, and constructed a sustainable theoretical model of bicycle sharing. Chen et al. [19] integrated the expectation confirmation theory with the technology acceptance model, and then incorporated marketing activities, conversion costs, and online public opinion into the model; in their empirical findings, it was found that technology acceptance, conversion costs, expected recognition, and marketing activities have a significant positive impact on the continuous use intention of the social media of mobile travel applications. 

In recent years, with the development of the economy and society, public health awareness has been significantly improved. Some scholars have studied the information level of health-related social media. Griffiths et al. [20] found, through empirical research, that a large number of patients with depression are more willing to search and obtain relevant health information from online forums and other social media; Li et al. [21] comparatively studied the factors affecting the health information search behavior of Italian and Chinese users on social media; Chen [22] constructed a user’s information search behavior model based on social media. The research of Gayle et al. [23] shows that college students are more willing to pay attention to, and obtain, health information through social media. Huang et al. [24] also found that young adults pay great attention to the health information or health status published by their friends on social media. Jin [25] et al. studied the influence mechanism of the WeChat users of social media spreading health information in the circle of friends; Nainggolan et al. [26] found that doctors in the medical school of the University of Malaysia would choose to use social media to obtain health knowledge and enhance their popularity. Zhang et al. [27] found, through a survey of 76 Chinese young adults living in the United States, that most young adults will spread health information through well-known social media at home and abroad. Other scholars have carried out research based on the social support level of health-related social media. Hobbs et al. [28] found that there is a significant positive relationship between users’ health status and the use of online social media. Online social media provides users with social support and encourages users’ healthy social behavior; Shaw et al. [29] study found that social media is conducive to promoting positive health behaviors of patients with diabetes. Naslund et al. [30] also showed that patients with mental illness can obtain emotional and social support through social media, thus promoting the treatment of the disease [31,32].

Through systematic combing and analysis, we found that the existing literature has conducted preliminary research on the continuous use intention of health-related social media, but there are still the following areas that can be further studied: At present, there is little literature on the effect of service quality on the continuous use intention of health-related social media. Existing documents rarely reveal the mechanical process of the continuous use intention of health-related social media, in particular, and there are few studies on how young adults’ emotional experiences (such as pan-family consciousness) and psychology change in online communities, and how emotional experience or psychological state affects the continuous use intention of health-related social media. Based on the SOR theory, this paper constructs a mechanical model of the continuous use intention of health-related social media, and studies the effects of factors such as service quality, emotional support, and information support on pan-family consciousness, as well as the influence mechanism of pan-family consciousness on the continuous use intention of health-related social media.

## 3. Theoretical Framework and Hypotheses Development

**Figure 1:**
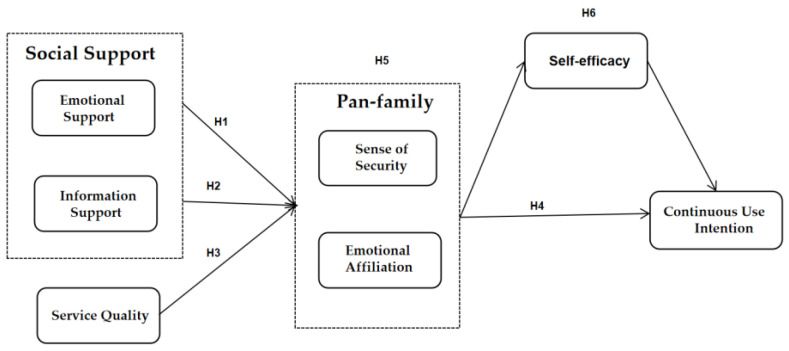
Conceptual model.

The research model of this paper is shown in Figure 1. Social support and service quality refers to “stimulus” (S) factors, and social support includes information support and emotional support. As the inner activity (O) of “organism”, pan-family consciousness includes two factors, namely, sense of security and emotional affiliation. Pan-family consciousness affects the continuous use intention of health-related social media (R).

Based on the theory of social exchange, Crocker [51] proposed a model of the influence mechanism of online social support on customer citizenship behavior, which confirmed that online social support can be expressed in the following two dimensions: informational support and emotional support. Based on this, this paper believes that social support includes two dimensions, namely, emotional support and information support. Emotional support is mainly manifested in the care, comfort, encouragement, etc., that young adults receive on health-related social media, from other young adults. Young adults on health-related social media understand each other through health-related social media communication and interaction; the care of friends provides strong emotional support, and empathetic young adults resonate emotionally. A higher level of emotional support is more helpful for young adults to carry out continuous exchanges in health-related social media, and makes young adults more dependent on other members of said health-related social media [52]. Pan-family consciousness refers to the fact that health-related social media members regard social media platforms such as QQ and WeChat as a home, regard social media members as family members, and actively share and exchange health information on social media platforms. Lin [53] called the support that young adults received on health-related social media, such as encouragement, respect, and comfort, social utility, and this support significantly affects a young adult’s sense of belonging in the pan-family consciousness. For example, NetEase Cloud Music gives full play to the role of music opinion leaders, taking music socialization as an entry point, providing young adults with health-related social media to exchange music; it also allows many young adults to tell their stories in the form of music reviews and to accept the comfort and encouragement of other members on the health-related social media, so as to have a stronger sense of dependence on this health-related social media. Therefore, the following hypotheses can be made:

**Hypothesis** **1** **(H1).** *Emotional support will significantly affect pan-family consciousness*;

**Hypothesis** **1a** **(H1a).** *Emotional support will significantly affect the sense of security*;

**Hypothesis** **1b** **(H1b).** *Emotional support will significantly affect the emotion affiliation*.

Information support refers to information that can help solve problems, such as opinions, suggestions, and countermeasures [54]. The information processing theory believes that people cannot often evaluate information, so information support effectively promotes young adults to make relevant decisions. On social media, the platform also provides effective information support, such as Weibo Interactive, WeChat Business, Taobao, and other platforms reviews, as well as live broadcasts, buyer shows, seller shows, and other information, which provide a reference for young adults and help young adults make consumption decisions. At the same time, social media also provide young adults with a sharing platform [55]; as members continue to interact, they establish a deeper understanding, and when members are interested in a topic or information on the health-related social media, they invest more time and energy to further develop a certain family-like dependence on the health-related social media. The research of Blanchard [56] shows that the information exchange and information swap between members enhance the trust of others and the perception of their value; the stronger this trust and perception of self-worth, the stronger the pan-family consciousness will be. Huang et al. [57] also pointed out in their research that the barrage of information delivered immediately provides young adults with a good sense of immersion, and has a significant impact on the participation behavior of young adults. For example, webcasting frees young adults from passive viewing habits. Young adults and anchors can exchange and transmit information through barrage form, so the audience stickiness of webcasts is much higher than that of traditional media. Therefore, good information support can enable young adults to quickly integrate into their health-related social media, and generate a strong sense of pan-family. Therefore, the following hypotheses can be made:

**Hypothesis** **2** **(H2).** *Information support will significantly affect pan-family consciousness*;

**Hypothesis** **2a** **(H2a).** *Information support will significantly affect the sense of security*;

**Hypothesis** **2b** **(H2b).** *Information support will significantly affect the emotion affiliation*.

In terms of technology, a health-related social media platform as an information system [58], and its page design, system stability, content, etc., directly affect young adult experience and identity with the health-related social media, as well as determine whether young adults are willing to continue using the health-related social media. According to the success model of information systems, service quality is one of the key success factors of information systems. Therefore, based on this model, this paper examines the effect of health-related social media service quality on the continuous use intention of health-related social media. Service quality reflects the reliability, empathy, guarantee, and timeliness of the services provided by health-related social media [59]. Alali and Salim [60] pointed out that service quality has a significant positive impact on young adult satisfaction. Liang [61] and others also pointed out that high service quality can enable young adults to achieve their expectations, and thus generate satisfaction with health-related social media. If health-related social media can provide young adults with reliable and guaranteed services, and can solve the problems encountered by young adults on time, it indicates that the health-related social media has high service quality and is worthy of young adults’ trust—that is, it can make young adults agree from the heart. Moreover, if health-related social media can start from the needs of young adults and provide young adults with professional services, it enables young adults to cultivate good usage habits, and to have a strong sense of dependence and belongingness to said health-related social media, thereby affecting pan-family consciousness. Therefore, this paper assumes the following:

**Hypothesis** **3** **(H3).** *Service quality will significantly affect pan-family consciousness*;

**Hypothesis** **3a** **(H3a).** *Service quality will significantly affect the sense of security*;

**Hypothesis** **3b** **(H3b).** *Service quality will significantly affect the emotional affiliation*.

Zhao [62] and other studies have shown that if young adults have a strong sense of belongingness to a certain health-related social media, they will slowly recognize the normative system of the health-related social media and accept the opinions of other young adults. Therefore, they will be more willing to participate in the exchanges and activities of health-related social media. Kim et al. [63] believe that when young adults’ sense of membership increases, they regard themselves and other members as one, and are willing to invest more time and energy into health-related social media. Tonteri et al. [64] pointed out that young adults participate in online communities to meet their entertainment, social, or functional needs. If these needs are met, they will use health-related social media more. At the same time, if a member has continuous influence in the health-related social media, the young adult will become an opinion leader and will feel more of a sense of accomplishment and pride, and his comments will also affect more health-related social media participants. In addition, the sustainable development of health-related social media benefits from the strong emotional connection between young adults, that is, the emotional connection between young adults and communities, and between young adults themselves, has a positive impact on young adult participation. Therefore, pan-family consciousness can promote the continuous use intention of health-related social media. Based on this, this paper proposes the following hypotheses:

**Hypothesis** **4** **(H4).** *Pan-family consciousness will significantly affect the continuous use intention of health-related social media*;

**Hypothesis** **4a** **(H4a).** *Sense of security will significantly affect the continuous use intention of health-related social media*;

**Hypothesis** **4b** **(H4b).** *Emotion affiliation will significantly affect the continuous use intention of health-related social media*.

Emotional support, information support, and service quality on health-related social media, mainly provide young adults with a service environment. In this environment, young adults can better seek help and support. Therefore, its impact on young adults’ continuous use of health-related social media is an indirect process. Moreover, China is a society with family as the link, the most important thing for people is family consciousness, and they often restrict their behavior by family social norms; therefore, when young adults are active on the health-related social media, they will also develop a sense of family for the health-related social media. Zhao [33] thinks that this special feeling for a family is “pan-family consciousness”, which will make them feel that they are part of the health-related social media, and they will participate in the activities of the health-related social media more actively, so as to make the use behavior more lasting. Moreover, emotional support, information support, and service quality all positively affect young adults’ pan-family consciousness. The pan-home consciousness is positively correlated with the continuous use intention of health-related social media. Therefore, this paper believes that emotional support, information support, and service quality will have a significant impact on the continuous use intention of health-related social media, through pan-family consciousness. Therefore, this paper assumes the following:

**Hypothesis** **5** **(H5).** *Pan-family consciousness plays an intermediary role in the impact of emotional support, information support, and service quality on the continuous use intention of health-related social media*;

**Hypothesis** **5a** **(H5a).** *Sense of security plays an intermediary role in the impact of emotional support, information support, and service quality on the continuous use intention of health-related social media*;

**Hypothesis** **5b** **(H5b).** *Emotion affiliation plays an intermediary role in the impact of emotional support, information support, and service quality on the continuous use intention of health-related social media*.

Self-efficacy is a concept proposed by Bandura, and is an important part of the theoretical system of Bandura’s Department of Sociology. He stated that the so-called self-efficacy refers to an individual’s expectation of whether they can complete a certain behavior in a specific situation. Hsu et al. [65] found that self-efficacy is an important determinant of the continuous use intention of health-related social media. Ma and Agarwal [66] and Ryan [67] also used empirical research to prove that in a health-related social media environment, self-efficacy has a direct impact on the continuous use intention of health-related social media. Some other scholars, for example, Lin [68] and Liao [69], have also confirmed that self-efficacy in health-related social media has a positive impact on the continuous use intention of health-related social media. At the same time, Bandura emphasized that self-efficacy is not the cause of people’s behavior, but only a moderating effect on people’s certain behavior and behavioral results. Scholars Svetlik et al. [70] investigated the moderating effect of self-efficacy on the continuous use intention of health-related social media by investigating and analyzing 445 health-related social media members. Hau et al. [71] also surveyed 213 mobile phone health-related social media members and successfully verified the moderating effect of self-efficacy on the continuous use intention of health-related social media. Therefore, most scholars have verified the regulating effect of self-efficacy. However, this paper believes that young adults with strong pan-family consciousness will also have a significant impact on the continuous use intention of health-related social media, through stronger self-efficacy, that is, pan-family consciousness will better affect the continuous use intention of health-related social media through the mediating effect of self-efficacy. Therefore, this paper assumes the following:

**Hypothesis** **6** **(H6).** *Self-efficacy plays a mediating role in the influence of pan-family consciousness on the continuous use intention of health-related social media*;

**Hypothesis** **6a** **(H6a).** *Self-efficacy plays a mediating role in the influence of a sense of security on the continuous use intention of health-related social media*;

**Hypothesis** **6b** **(H6b).** *Self-efficacy plays a mediating role in the influence of emotion affiliation on the continuous use intention of health-related social media*.

## 4. Research Methodology

### 4.1. Sample

This paper uses the questionnaire survey method to collect data and test hypothesis relationships. The questionnaire consists of the following three parts: a brief description of survey content, the items of each construct, and the basic personal information of the investigators. This paper uses the most authoritative online survey platform, “Questionnaire Star”, to collect data from Chinese young people. At the same time, the simple random sampling method, which is widely used in social surveys and social research, is adopted to ensure the representativeness of samples. A total of 440 questionnaires were collected, and after sorting out the questionnaires and deleting 26 identical and regular questionnaires, deleting 26 longer and shorter response time questionnaires, 388 valid questionnaires were retained, indicating an effective questionnaire response rate of 88%. Qualitative methods are very important, but for the current research topic, we feel that quantitative methods may be more specific. The detailed information of the valid questionnaire samples is shown in Table 1. With the increasing pressure on study, life and work, more and more young people in China will have some health puzzles, such as insomnia, obesity, headache, and psychological health problems. Young people growing up in the internet atmosphere are more likely to query, share and exchange health-related information through social media. Especially since the outbreak of COVID-19, people have paid more and more attention to health problems, and the frequency of access to health-related social media is increasing. As shown in Table 1, about 95% of the sample population is under 40 years old, so the overall sample is acceptable.

### 4.2. Measurement of the Variables

The questionnaire included basic information, including the measurement of the impact of social support and service quality on the continuous use intention of health-related social media. The social support scale mainly adopted the mature scales developed by Liang [61], Zhou [72], etc. The service quality scale was based on the scale adopted by Delone and Mclean [59], Todd [73], etc. The pan-family consciousness scale was based on the main scale content of Wachs [74], Albanesi et al. [75], Lin [53], and Zhao [33]. The self-efficacy scale used four measurement items formed by the scales of Hsu et al. [65] and Oliver [76]. The measurement of the continuity of usage behavior drew upon the measurement scales of Jun et al. [77] and Chen [44]. This information is shown in the appendix. The questionnaire included a 5-point Likert scale, with each point corresponding to either strongly disagree, disagree, general, agree, or strongly agree, allowing the respondents to choose according to their feelings and actual conditions.

### 4.3. Formatting of the Mathematical Components

Reliability essentially refers to whether the results of the measurement questionnaire are reliable, or whether the subjects answered the sample questions seriously. This paper used Cronbach’s α coefficient and composite reliability (CR) to detect whether the test results truly reflected the stable and consistent characteristics of the subjects. According to Table 2, the Cronbach’s α coefficient of the social support overall scale is 0.867 (>0.70) and 0.801 (>0.70), that of the service quality is 0.766 (>0.70), that of the pan-family consciousness scale is 0.911 (>0.70), that of the self-efficacy measurement scale is 0.845 (>0.70) and 0.849 (>0.70), and that of the self-efficacy measurement scale is 0.820 (>0.70). The α coefficients of the scales are all greater than 0.7, and the composite reliability (CR) of the scales are all greater than 0.7, which indicates that the internal consistency of the scale data is good.

Validity refers to the consistency between the test score and the expected score. In other words, it indicates whether the test score can reflect the characteristics it wants to measure. This paper selected common validity tests, namely, the Kaiser–Meyer–Olkin (KMO) value and the Bartlett sphere test. From the data, we can see that the KMO values of social support, service quality, pan-family consciousness, self-efficacy, and continuous use intention of health-related social media are 0.890 (>0.60), 0.700 (>0.60), 0.928 (>0.60), 0.794 (>0.60), and 0.698 (>0.60), respectively. Moreover, the *p*-value of the significance coefficient of the Bartlett sphere test of these scales is less than 0.001. Therefore, the scale passed the KMO value and Bartlett sphere tests, indicating that the validity of the structure of the scale is good. In addition, the most of standard factor loading is above 0.7, AVE value is more than 0.5, indicating that the scale has good validity. In terms of discriminant validity, if the correlation coefficient between latent variables is less than the square of AVE, it shows that the latent variables can be distinguished, as can be observed from Table 3, and if the correlation coefficient between variables is less than square of AVE, it shows that all variables have good discriminant validity, and it has passed the validity test.

Then, AMOS24.0 statistical software was used for confirmatory factor analysis. Several important fitting indexes of the model are as follows: CMIN = 713.49, DF = 278, CMIN/DF = 2.567, RMSEA = 0.064, GFI = 0.863, AGFI = 0.827, CFI = 0.928, NFI = 0.888. As shown in Table 4, most fitting indexes were higher than the minimum fitting standard values, indicating that the model fitted well.

### 4.4. Correlation Analysis

In order to understand the relationship between the various dimensions of social support, and service quality, pan-family consciousness, and the continuous use intention of health-related social media, this paper used correlation analysis to study whether there is a close relationship between the various variables in terms of development direction and size (as shown in Table 3).

The correlation coefficients between the two dimensions of social support and service quality in the sense of security in the pan-family consciousness are 0.699, 0.696, and 0.674, respectively, and the corresponding *p*-values of the correlation coefficients are all less than 0.01, proving that these dimensions have a significant positive correlation with the sense of security in the pan-family consciousness. The correlation coefficients between the two dimensions of social support and service quality with the emotional affiliation in pan-family consciousness are 0.674, 0.645, and 0.623, respectively, and the corresponding *p*-values of the correlation coefficients are all less than 0.01, proving that these dimensions have a significant positive correlation with the emotional affiliation in the pan-family consciousness. In summary, the stronger the social support and service quality perceived by the members of health-related social media, the higher their corresponding pan-family consciousness in the health-related social media will be.

The correlation coefficients between the two dimensions of sense of security and emotional affiliation in the pan-family consciousness with the continuous use intention of health-related social media are 0.678 and 0.693, respectively, and the corresponding *p*-values are all less than 0.01, showing that there is a significant positive correlation between pan-family consciousness and the continuous use intention of health-related social media. That is to say, the stronger the pan-family consciousness of the members of health-related social media, the stronger the continuous use intention of health-related social media.

### 4.5. Model Hypotheses Test

This paper used AMOS24.0 software to make path analysis and hypothesis tests of the research model. At the same time, regression analysis was used to analyze the mediating role of pan-family consciousness in emotional support, information support, service quality, and continuous use intention of health-related social media, and the mediating role of self-efficacy between pan-family consciousness and persistence of continuous use intention of health-related social media.

#### 4.5.1. Model Test

**Figure 2:**
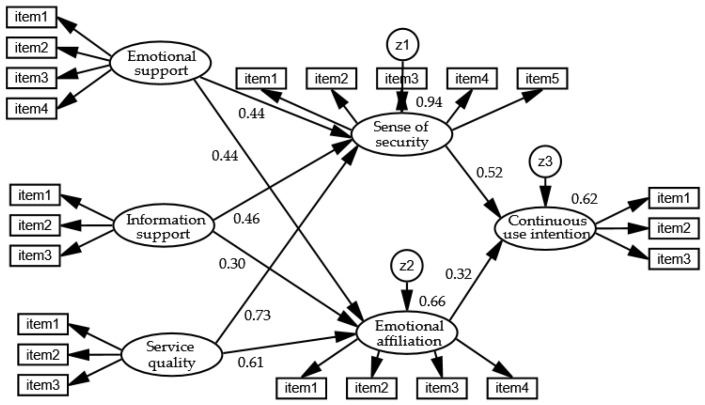
Path Coefficient.

Firstly, the fitness of the research model was tested, several important fitting indexes of the model test are as follows: CMIN = 1011.106, DF = 201, CMIN/DF = 5.03, RMSEA = 0.102, GFI = 0.804, AGFI = 0.753, CFI = 0.837, NFI = 0.806. According to Table 3, some indexes are higher than the minimum fitting standard, which indicates that the model fitting is general. The reason for the general fit of the model is that the data collected do not conform to the multivariate normal distribution (multivariate kurtosis and its critical ratio = 70.732, much higher than the standard of multivariate kurtosis and its critical ratio ≤ 5), and if the data do not conform to the multivariate normal, the chi-square value will expand and the fitting effect will be affected; however, the Bollen-Stine bootstrap can be used to fit statistic bias that occurs in structural equation modeling applications due to non-normal data [78,79]. Therefore, the Bollen-Stine can be used to modify the model. The fitting value of the modified model is as follows: CMIN = 304.536, DF = 201, CMIN/DF = 1.52, RMSEA = 0.04, GFI = 0.94, AGFI = 0.92, CFI = 0.98, NFI = 0.94, these fitting indexes were higher than the fitting standard values, indicating that the model fitted well. Then using AMOS software to test the significance of the path coefficient of the research model. Firstly, it examines the influence of emotional support, information support, and service quality on pan-family consciousness, and the influence of pan-family consciousness on the continuous use intention of health-related social media, as shown in Figure 2 and Table 5. Then Table 4 shows the estimated value and the *p*-value of the standardized path coefficients of the structural relationship between the latent variables. The results show that information support and emotional support have a significant positive impact on pan-family consciousness, and the standardized path coefficients of the impact on security are 0.435 and 0.465, respectively; the influence standardized path coefficients on emotional affiliation are 0.443 and 0.296, respectively. Service quality has a significant positive impact on the sense of security and emotional affiliation, and the standardized path coefficients are 0.728 and 0.611, respectively. Finally, the stronger the young adult’s pan-family consciousness of the health-related social media, the stronger the continuous use intention of health-related social media, and the standardized path coefficients of security and emotional affiliation are 0.517 and 0.318, respectively. Therefore, the hypotheses H1a, H1b, H2a, H2b, H3a, H3b, H4a, and H4b have been verified.

#### 4.5.2. Regression Analysis

In order to study the mediating effect of pan-family consciousness in social support and service quality on the continuous use intention of health-related social media, this paper uses SPSS to conduct a hierarchical regression analysis. To prove the mediating effect of pan-family consciousness, the following three conditions must be met: First, emotional support, information support, and service quality have a significant impact on the continuous use intention of health-related social media. Second, emotional support, information support, and service quality have a significant impact on pan-family consciousness. Third, when emotional support, information support, service quality, and pan-family consciousness have a hierarchical regression analysis on the effect of continuous use intention of health-related social media, emotional support, information support, and service quality will be disturbed by the pan-family consciousness, and the effect on the continuous use intention of health-related social media will be weakened.

The regression results are shown in Table 6. In the first model, the adjusted R-squared value is 0.507. Through the regression equation (*F* = 133.442, *p*-value < 0.01) test, the explanatory degree of the independent variable reaches 51%, indicating that the established regression model has good goodness of fit; it is proved that emotional support, information support, and service quality will have a significant impact on the continuous use intention of health-related social media, and condition one is met. In the second model and the fourth model, the adjusted R-squared values are 0.650 and 0.574. Through the regression equation (*F* = 240.181, 174.708, *p*-value < 0.01), the explanatory degrees of the independent variables reached 65% and 57%, indicating that the established regression model has good goodness of fit; it proves that emotional support, information support, and service quality have a significant impact on pan-family consciousness, and condition two is met. In model 3 and model 5, when the sense of security and emotional affiliation are both mediating variables, the regression coefficients of information support and service quality on the continuous use intention of health-related social media are all reduced, and the *p*-value is still less than 0.01. Therefore, it is believed that under the intermediary effect of pan-family, the influence of information support and service quality on the continuous use intention of health-related social media is still significant. Although the coefficient of emotional support in the regression model has declined, the *p*-value is greater than 0.1, the regression model is not significant. Therefore, the pan-family consciousness plays a part of the mediating role in the influence of information support and service quality on the continuous use intention of health-related social media, but does not play a mediating role in the influence of emotional support on the continuous use intention of health-related social media. Therefore, hypotheses H5a and H5b are partially established.

Then this article verifies the mediating role of self-efficacy; to prove that self-efficacy plays an intermediary role in the continuous influence of pan-family consciousness on the continuous use intention of health-related social media, three conditions need to be met. First, the pan-family consciousness has a significant impact on the continuous use intention of health-related social media. Second, the pan-family consciousness has a significant impact on self-efficacy. Third, in the hierarchical regression analysis of the effect of pan-family consciousness and self-efficacy on the continuous use intention of health-related social media, pan-family consciousness is disturbed by self-efficacy, and its influence on the continuous use intention of health-related social media is weakened.

The results are shown in Table 7. In model 6 and model 9, using the sense of security and emotional affiliation as independent variables, the dependent variable as the continuous use intention of health-related social media regression analysis, *F*-value is 328.627 and 356.812, and *p*-value < 0.01, the regression equation test shows that the regression model has good goodness of fit; it proves that pan-family consciousness has a significant impact on the continuous use intention of health-related social media, and the condition is met. In model 7 and model 10, the regression coefficients of sense of security and emotional affiliation are both significant at the level of 0.01, and the *F*-values are 470.879 and 465.220, indicating that the established regression model has a good fit. It proves that pan-family consciousness has a significant impact on the continuous use intention of health-related social media, and condition two is satisfied. In model 8 and model 11, when self-efficacy is the intermediary variable, the regression coefficients of sense of security and emotional affiliation to the continuous use intention of health-related social media both decrease, and the *p*-value is still less than 0.01; therefore, it is believed that under the mediating effect of self-efficacy, the effect of sense of security and emotional affiliation on the continuous use intention of health-related social media is still significant, condition three is established. Therefore, self-efficacy plays a partially mediating role in the influence of pan-family on the continuous use intention of health-related social media. Hypotheses H6a and H6b are established.

## 5. Conclusions

Based on the literature analysis and theoretical review, this paper constructed research hypotheses based on the SOR theoretical framework, mainly explored the theoretical model of the influencing factors of the continuous use intention of health-related social media, and verified the research hypotheses by way of questionnaires and empirical analysis. The results and discussion are as follows:

(1) Emotional support and information support have a significant positive impact on pan-family consciousness, indicating that the stronger the social support, the more health-related social media can make young users feel at home. Suppose that H1 and H2 are true. This shows that an important factor supporting young adult’s use of health-related social media, is social support. Young adult users can communicate and interact with friends, and even strangers, through the social function of health-related social media; they can also gain more information through health-related social media, to improve health awareness. However, the influence of information support (path coefficient of 0.465) on sense of security is stronger than that of emotional support (path coefficient of 0.435). Because most of the young adults on health-related social media exchange information with each other, when they encounter difficulties, they gain information and suggestions from other members of the health-related social media, so the influence of information support on the sense of security is more obvious. However, the difference in path coefficients between emotional support and information support is not very large, which shows that the influence of emotional support on young adults is equally important. Emotional support is conducive to deepening the emotional communication among health-related social media members, and enhancing the sense of identity of said health-related social media. In addition, the influence of emotional support (path coefficient of 0.443) on emotion affiliation is stronger than that of information support (path coefficient of 0.296). This is because emotional support can make young adults feel more support and encouragement, which has a positive impact on the emotion affiliation. The more emotional support young adults receive from health-related social media, the stronger their emotional affiliation will be. Therefore, health-related social media can pay attention to the emotional needs of young adults, so as to better promote the development of health-related social media.

(2) The results show that service quality has a significant effect on pan-family consciousness, indicating that good service can reduce the effort costs of young adults and can optimize the young adult experience [80]. Suppose H3 holds. Compared with professional hospitals or other organizations, the convenience, immediacy, and freedom from the space and time of health-related social media promote young users to choose to use health-related social media. Moreover, the path coefficient of service quality is higher than that of social support, which indicates that the influence of service quality on pan-family consciousness is stronger than that of social support. Take a microblog group as an example, it can always get many young adult comments, clicks, and forwarding according to the current hot health topics, and it provides more personalized and intimate health services, so that the group young adults can find their health topics of interest and can actively participate in it. Therefore, maintaining the continuous use intention of health-related social media is an important way for the sustainable development of health-related social media.

(3) Pan-family consciousness positively affects the continuous use intention of health-related social media. That is, the stronger the pan-family consciousness, the stronger the young users’ intention to continue to use health-related social media. Suppose H4 holds. Because the family has always been the most intimate link among Chinese people, when members of health-related social media have a strong pan-family consciousness of said health-related social media, they inevitably regard the health-related social media as a big family. Every member of the health-related social media is a member of the family, so they will continue to use the health-related social media. Therefore, pan-family consciousness positively affects the continuous use intention of health-related social media.

(4) This paper verifies that pan-family consciousness plays a part of the intermediary role in information support, service quality, and continuous use intention of health-related social media, but not in emotional support and the continuous use intention of health-related social media. As far as the results are concerned, emotional support has no significant effect on the continuous use intention of health-related social media. Under the mediating effect of pan-familial consciousness, the impact is still not significant, so it is believed that pan-familial consciousness does not play a mediating role between the two. Information support and service quality have a positive impact on the continuous use intention of health-related social media. Under the mediating effect of pan-family consciousness, the impact is still significant, and the regression coefficients of emotional support and service quality are lower than the regression coefficients of emotional support and service quality, directly affecting the continuous use intention of health-related social media. So, this paper believes that pan-family consciousness plays a part of the mediating role between information support, service quality, and the continuous use intention of health-related social media. It is assumed that H5 was partially verified. This is because, compared with emotional support, information support and service quality are the main advantages of health-related social media, and they are also important reasons for young users to use health-related social media. Therefore, health-related social media should focus on information support and service quality when creating a sense of home for young users.

(5) Self-efficacy plays a partial mediating role in pan-family consciousness and the continuous use intention of health-related social media. In terms of results, pan-family consciousness has a positive impact on the continuous use intention of health-related social media; under the mediating effect of self-efficacy, the impact is still significant, and the regression coefficient of pan -family consciousness is lower than that in model 8, directly affecting the continuous use intention of health-related social media. In other words, the pan-family consciousness has a certain degree of influence on the continuous use intention of health-related social media, through self-efficacy. Therefore, this paper believes that self-efficacy plays a part in the mediating role between pan-family consciousness and the continuous use intention of health-related social media. It is assumed that H6 was partially verified. This is mainly because when young users of health-related social media experience the feeling of home, they may stimulate a stronger sense of self-ability, so as to promote their willingness to continue to use health-related social media.

## 6. Implications

In order to better promote young adults sustainable use intention of health-related social media, this paper obtains the following enlightenment, according to the research conclusions:

(1) Health-related social media should improve their service quality. Service quality has a positive impact on young adult’s perception of pan-family consciousness. Therefore, health-related social media managers should pay more attention to the personalized health needs of young users in their daily operation and management, and enhance the sense of belonging of young users, by improving their user experience. At the same time, they should improve and optimize the service system of health-related social media, ensure the service quality of health-related social media, and make health-related social media members feel at home all the time.

(2) Health-related social media should pay attention to young adult’s pan-family consciousness. If the members of health-related social media are very close, members will feel that health-related social media is a home, and the emotional belonging among the health-related social media members will be closer. The health-related social media members will be more willing to share and exchange health information and will form the habit of continuous use. Health-related social media managers can take some measures, such as initiating some hot health-related discussion topics, to involve young users to arouse resonance. Some interesting health activities or lectures can also be held. Through these exchanges and interactions, young users can have a sense of home, to enhance the contact between the members of the healthy social media, so as to enhance the willingness to continue to use healthy social media. In addition, a good atmosphere could be created and young users could be encouraged to actively interact with each other in emotion and information. Family culture can also be introduced into health-related social media, and some health-related activities similar to family gatherings can be held, so as to create a good group atmosphere, enhance young adults’ identity with health-related social media, and increase their willingness to continue to use them.

(3) Self-efficacy can make pan-family consciousness better affect the willingness to use health-related social media. Therefore, health-related social media should pay attention to the mediating role of self-efficacy. The stronger the self-efficacy of adolescents, the easier it is to actively and optimistically participate in the interaction and communication of health-related social media. Some young adults with outstanding abilities will also become social media leaders, to better lead other members to maintain their willingness to continue to use the health-related social media, so as to promote the virtuous circle of the whole health-related social media system.

## 7. Limitations and Future Research

Based on the perspective of Chinese culture, this paper constructed a theoretical model of the influencing factors of the continuous use intention of health-related social media. The research conclusion has a certain reference value for the sustainable development of health-related social media. However, due to various reasons, this study has certain limitations. The shortcomings of this paper and the direction of improvement in the future are as follows:

(1) Most of the respondents in this paper were young people aged 19–30. Although they are an important young adult population, a larger range of samples needs to be investigated in future research, so as to enhance the universality of the research results.

(2) The antecedents of pan-family consciousness are various and complex. This paper only considered service quality and social support, but not the young adults’ factors. Therefore, in future research, the similarity in perception and individual cognition should be considered.

(3) In addition to pan-family consciousness, there may be other factors affecting the continuous use intention of health-related social media. Future research should also explore the impact of these factors.

## Figures and Tables

**Table 1:** Basic information of the survey samples (*n* = 388).

Sample Feature		Number of Persons	Percentage
Gender	Male	180	46.4%
	Female	208	53.6%
Age	19–24 years old	219	56.4%
	25–30 years old	71	18.3%
	31–35 years old	55	14.2%
	36–40 years old	24	6.2%
	41–45 years old	9	2.3%
	45 years old and above	10	2.6%
Education level	High school/technical secondary school and below	10	2.6%
	Junior college	29	7.5%
	Undergraduate	333	85.8%
	Master’s degree and above	16	4.1%
Frequency of visiting health-related social media	Every day	298	76.8%
	2–3 days	48	12.4%
	4–5 days	23	5.9%
	6–7 days	8	2.1%
	More than 7 days	11	2.8%
Time to join health-related social media	1 year and less	29	7.5%
	1–2 years	84	21.6%
	2–3 years	82	21.1%
	3–4 years	49	12.6%
	4 years and above	144	37.1%

**Table 2:** Standard factor loading and reliability test.

Variable	Item	Standard Factor Loading	α	CR	AVE
ES	ES1	0.679	0.867	0.871	0.629
	ES2	0.818			
	ES3	0.824			
	ES4	0.840			
IS	IS1	0.780	0.801	0.801	0.573
	IS2	0.759			
	IS3	0.731			
SQ	SQ1	0.752	0.766	0.769	0.527
	SQ2	0.670			
	SQ3	0.753			
SOS	SOS1	0.750	0.845	0.847	0.525
	SOS2	0.699			
	SOS3	0.706			
	SOS4	0.734			
	SOS5	0.732			
EA	EA1	0.775	0.849	0.849	0.584
	EA2	0.761			
	EA3	0.753			
	EA4	0.766			
SE	SE1	0.769	0.820	0.822	0.537
	SE2	0.716			
	SE3	0.694			
	SE4	0.749			
CUI	CUI1	0.760	0.787	0.791	0.558
	CUI2	0.715			
	CUI3	0.765			

**Table 3:** Correlation coefficient matrix and AVE square root.

	Emotional Support	Information Support	Service Quality	Sense of Security	Emotional Affiliation	Self-Efficacy	Continuance Usage Intention
Emotional support	0.933						
Information support	0.644 **	0.895					
Service quality	0.525 **	0.599 **	0.877				
Sense of security	0.699 **	0.696 **	0.674 **	0.920			
Emotional affiliation	0.674 **	0.645 **	0.623 **	0.835 **	0.921		
Self-efficacy	0.613 **	0.663 **	0.613 **	0.741 **	0.739 **	0.907	
Continuous use intention	0.543 **	0.642 **	0.623 **	0.678 **	0.693 **	0.744 **	0.889

Note: ** = being significant at or above 0.01. The data on the diagonal are the square root value of the AVE of the variable.

**Table 4:** Recommended value of the model fitting index.

Fitting Index	Recommended Value
CMIN/DF	<3
GFI	>0.90
AGFI	>0.90
CFI	>0.90
NFI	>0.90
RMSEA	<0.08

**Table 5:** Significance test of path coefficients.

			NSEPC	S.E.	C.R.	*p*	SEPC
Sense of security	<---	Emotional support	0.295	0.048	6.209	***	0.435
Emotion affiliation	<---	Emotional support	0.588	0.131	4.490	***	0.443
Sense of security	<---	Information support	0.312	0.051	6.121	***	0.465
Emotion affiliation	<---	Information support	0.230	0.063	3.634	***	0.296
Sense of security	<---	Service quality	0.548	0.064	8.578	***	0.728
Emotion affiliation	<---	Service quality	0.533	0.075	7.141	***	0.611
Continuous use intention	<---	Sense of security	0.588	0.131	4.490	***	0.517
Continuous use intention	<---	Emotion affiliation	0.313	0.120	2.610	**	0.318

Note: “NSEPC” = non-standardized estimated path coefficients; “SEPC” = standardized estimated path coefficients; *** = being significant at or above 0.001. ** = being significant at or above 0.01. Values inside brackets correspond to the C.R. value, i.e., *t* value.

**Table 6:** Analysis of the mediating role of pan-family consciousness.

Model	Mode1 1		Model 2		Model 3		Model 4		Model 5	
Dependent Variable	Y = Continuance Usage Intention		M1 = Sense of Security		Y = Continuous Use Intention		M2 = Emotion Affiliation		Y = Continuous Use Intention	
	B	SE	B	SE	B	SE	B	SE	B	SE
X1 = Emotional support	0.089 ***	0.034	0.381 ***	0.045	0.012	0.035	0.527 ***	0.064	−0.11	0.034
X2 = Information support	0.345 ***	0.051	0.414 ***	0.069	0.261 ***	0.052	0.447 ***	0.098	0.260 ***	0.049
X3 = Service quality	0.352 ***	0.046	0.537 ***	0.062	0.244 ***	0.049	0.617 ***	0.088	0.235 ***	0.046
M1 = Sense of security					0.202 ***	0.037				
M2 = Emotion affiliation									0.190 ***	0.025
R^2^	0.510		0.652		0.546		0.577		0.575	
Adjusted R^2^	0.507		0.650		0.541		0.574		0.570	
F	133.442 ***		240.181 ***		115.176 ***		174.708 ***		129.476 ***	

Note: *** = being significant at or above 0.001.

**Table 7:** Analysis of the mediating role of self-efficacy.

Model	Model 6		Mode7		Model 8		Model 9		Model 10		Model 11	
Dependent Variable	Y = Continuance Usage Intention		M = Self-Efficacy		Y = Continuous Use Intention		Y = Continuance Usage Intention		M = Self-Efficacy		Y = Continuous Use Intention	
	B	SE	B	SE	B	SE	B	SE	B	SE	B	SE
X1 = Sense of security	0.428 ***	0.024	0.583 ***	0.027	0.177 ***	0.031						
X2 = Emotion affiliation							0.337 ***	0.018	0.448 ***	0.021	0.153 ***	0.023
M = self-efficacy					0.430 ***	0.039					0.410 ***	0.038
R^2^	0.460		0.550		0.589		0.480		0.547		0.599	
Adjusted R^2^	0.458		0.548		0.587		0.479		0.545		0.597	
F	328.627 ***		470.879 ***		276.118 ***		356.812 ***		465.220 ***		287.263 ***	

Note: *** = being significant at or above 0.001.

## Data Availability

The data presented in this study are available on request from the corresponding author. The data are not publicly available due to ethical restrictions.
